# Early transcriptional and epigenetic divergence of CD8^+^ T cells responding to acute versus chronic infection

**DOI:** 10.1371/journal.pbio.3001983

**Published:** 2023-01-30

**Authors:** Lauren K. Quezada, Wenhao Jin, Yi Chia Liu, Eleanor S. Kim, Zhaoren He, Cynthia S. Indralingam, Tiffani Tysl, Lara Labarta-Bajo, Ellen J. Wehrens, Yeara Jo, Katelynn R. Kazane, Christopher Hattori, Elina I. Zuniga, Gene W. Yeo, John T. Chang

**Affiliations:** 1 Department of Medicine, University of California San Diego, La Jolla, California, United States of America; 2 Department of Cellular and Molecular Medicine, University of California San Diego, La Jolla, California, United States of America; 3 Division of Biological Sciences, University of California San Diego, La Jolla, California, United States of America; 4 Institute for Genomic Medicine, University of California San Diego, La Jolla, California, United States of America; 5 Department of Medicine, Jennifer Moreno Department of Veteran Affairs Medical Center, San Diego, California, United States of America; Children’s Hospital of Philadelphia and The University of Pennsylvania School of Medicine, UNITED STATES

## Abstract

During a microbial infection, responding CD8^+^ T cells give rise to effector cells that provide acute host defense and memory cells that provide sustained protection. An alternative outcome is exhaustion, a state of T cell dysfunction that occurs in the context of chronic infections and cancer. Although it is evident that exhausted CD8^+^ T (T_EX_) cells are phenotypically and molecularly distinct from effector and memory CD8^+^ T cells, the factors regulating the earliest events in the differentiation process of T_EX_ cells remain incompletely understood. Here, we performed single-cell RNA-sequencing and single-cell ATAC-sequencing of CD8^+^ T cells responding to LCMV-Armstrong (LCMV-Arm) or LCMV-Clone 13 (LCMV-Cl13), which result in acute or chronic infections, respectively. Compared to CD8^+^ T cells that had undergone their first division in response to LCMV-Arm (Div1_ARM_) cells, CD8^+^ T cells that had undergone their first division in response to LCMV-Cl13 (Div1_CL13_) expressed higher levels of genes encoding transcription factors previously associated with exhaustion, along with higher levels of Ezh2, the catalytic component of the Polycomb Repressive Complex 2 (PRC2) complex, which mediates epigenetic silencing. Modulation of Ezh2 resulted in altered expression of exhaustion-associated molecules by CD8^+^ T cells responding to LCMV-Cl13, though the specific cellular and infectious contexts, rather than simply the level of Ezh2 expression, likely determine the eventual outcome. Taken together, these findings suggest that the differentiation paths of CD8^+^ T cells responding to acute versus chronic infections may diverge earlier than previously appreciated.

## Abstract 2

How early can the process of T cell exhaustion begin? This study shows that CD8+ T cells that have undergone their first cellular division in response to chronic versus acute infection may have already diverged along transcriptionally and epigenetically distinct differentiation paths.

## Introduction

During a microbial infection, naïve CD8^+^ T cells undergo activation, expansion, and differentiation, giving rise to effector cells that provide acute host defense and memory cells that provide sustained protection [1]. Effector T cells secrete inflammatory cytokines such as interferon gamma (IFNγ) and tumor necrosis factor (TNF), along with cytolytic granules, such as granzymes and perforin, to kill infected cells. Memory T cells are long-lived cells that serve to provide a rapid response upon reinfection. However, an alternative outcome to the generation of effector and memory cells is exhaustion, which occurs in the setting of chronic infections and tumors, resulting in T cells that exhibit reduced function [2–4]. Lymphocytic choriomeningitis virus (LCMV) is a well-characterized model system that has been extensively used to study CD8^+^ T cell responses to acute and chronic infections. Acute infection with the LCMV-Armstrong (LCMV-Arm) strain results in viral clearance and generation of effector and memory T cells. By contrast, chronic infection with the LCMV-Clone 13 (LCMV-Cl13) strain, which differs from LCMV-Arm by two amino acids, results in persistent antigen and T cell exhaustion. In general, exhausted CD8^+^ T (T_EX_) cells exhibit increased expression of inhibitory receptors such as PD1, LAG3, TIM3, CTLA4, and TIGIT; reduced proliferative capacity when restimulated; and reduced cytokine production and function [3–5].

Compared to effector and memory T cells, T_EX_ cells exhibit an altered transcriptional program involving multiple transcription factors, including TOX, NFAT, IRF4, BATF, and NR4A family members, as well as a unique epigenetic landscape [6–14]. T_EX_ cells are heterogeneous and have been subdivided into at least three states: a progenitor or precursor state characterized by high expression of TCF1, SLAMF6, and CXCR5, along with low levels of T-bet; an intermediate or transitory state characterized by high levels of T-bet, CX3CR1, TIM3, and low levels of Eomes; and a terminal state characterized by high expression of TIM3, CD101, and Eomes, along with low levels of T-bet [15–21]. However, the timing and precise sequence of events regulating T cell exhaustion remain incompletely understood [22]; in particular, do T_EX_ cells transit from a functional effector state prior to commencing the exhaustion program, or can they bypass a functional intermediate effector state soon after initial activation?

Here, we performed single-cell RNA-sequencing (scRNA-seq) of CD8^+^ T cells responding to LCMV-Arm or LCMV-Cl13 at multiple time points following infection. Strikingly, CD8^+^ T cells that had undergone their first division in response to LCMV-Arm (“Div1_ARM_”) clustered distinctly from Div1 cells responding to LCMV-Cl13 (“Div1_CL13_”). Compared to Div1_ARM_ cells, Div1_CL13_ cells expressed higher levels of genes encoding transcription factors, including NFATC1 and NFATC2, that have been previously associated with exhaustion [9], along with Ezh2, the catalytic component of the Polycomb Repressive Complex 2 (PRC2) complex that mediates epigenetic silencing [23,24]. Moreover, Div1_CL13_ cells exhibited heterogeneity on the basis of their chromatin accessibility patterns. Modulation of Ezh2 by genetic deletion or retroviral overexpression approaches resulted in decreased or increased expression, respectively, of exhaustion-associated molecules by CD8^+^ T cells responding to LCMV-Cl13, though it should be noted that the specific cellular and infectious contexts, rather than simply the level of Ezh2 expression, likely determine the eventual outcome. Taken together, these findings indicate that the differentiation paths of CD8^+^ T cells responding to acute versus chronic infections may diverge earlier than previously appreciated.

## Results

### CD8^+^ T cells that have undergone their first division in response to acute versus chronic infection exhibit phenotypic, transcriptional, and epigenetic heterogeneity

**Fig 1:**
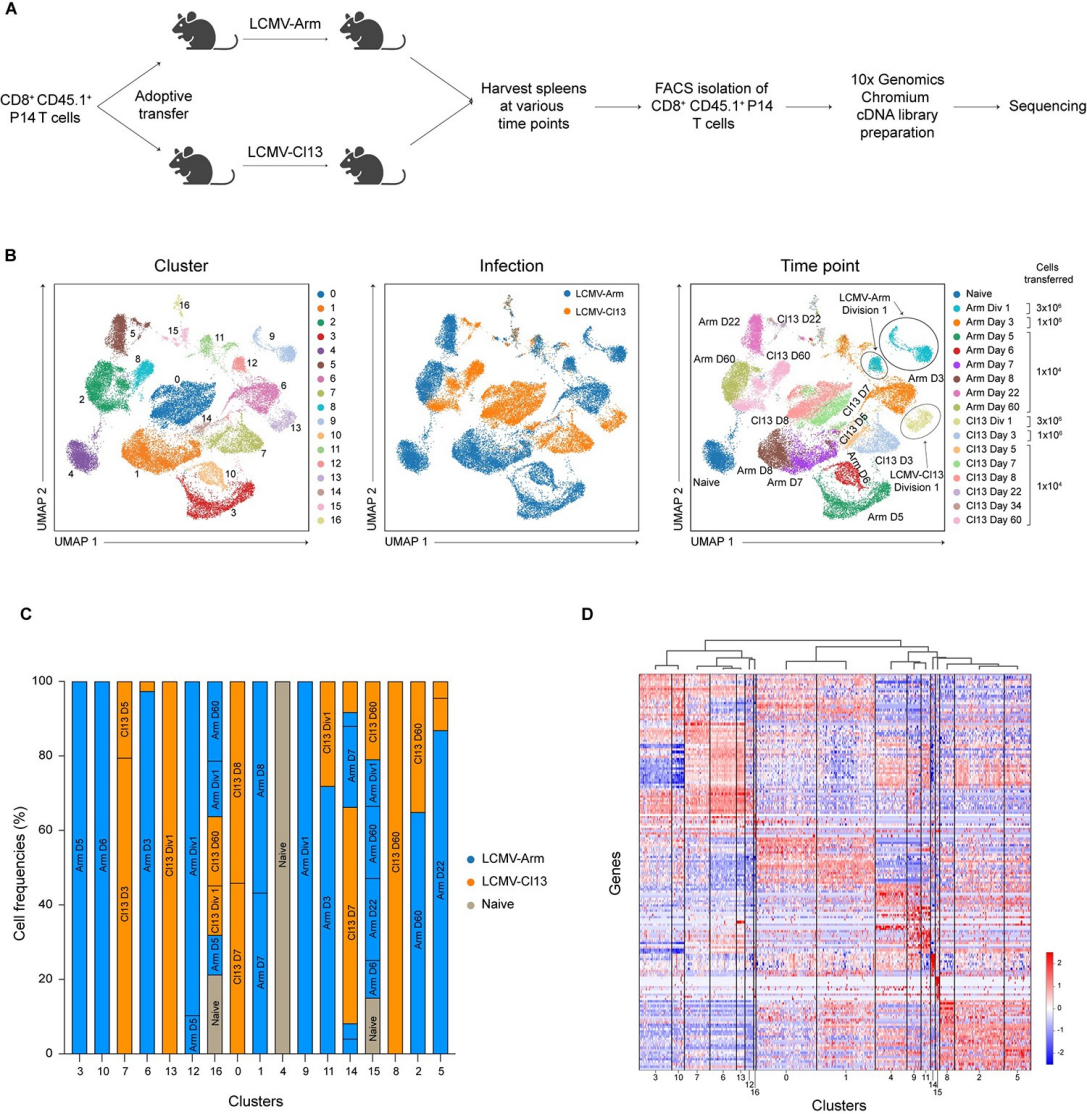
scRNA-seq analyses of CD8^+^ T cells responding to acute vs. chronic infection. (**A**) Experimental setup. CD8^+^CD45.1^+^ P14 T cells were adoptively transferred into separate CD45.2^+^ hosts 1 day prior to infection with either LCMV-Arm or LCMV-Cl13. To identify cells that had undergone their first division, some cells were labeled with CFSE prior to adoptive transfer. Splenocytes were harvested at the indicated time points after infection. Donor P14 CD8^+^ T cells were FACS-isolated and processed for scRNA-seq using the 10x Genomics Chromium platform. (**B**) UMAP clustering of all CD8^+^ cells, colored by cluster identity (left), infection type (middle), or time point (right); the three Division 1 clusters are circled for emphasis. (**C**) Bar graphs indicating the infection type and time point from which cells derived from each cluster are derived; clusters are grouped according to similarity in gene expression based on (D). (**D**) Hierarchical clustering of clusters based on gene expression profiles. The raw data for the panels in this figure are located in S1 Data file. Fig 1A created with BioRender.com. CFSE, carboxyfluorescein succinimidyl ester; LCMV-Arm, LCMV-Armstrong; LCMV-Cl13, LCMV-Clone 13; scRNA-seq, single-cell RNA-sequencing; UMAP, Uniform Manifold Approximation and Projection.

In order to compare CD8^+^ T cells responding to acute versus chronic infection, CD8^+^CD45.1^+^ P14 T cells, which have transgenic expression of a T cell receptor (TCR) that recognizes an immunodominant epitope of LCMV, were adoptively transferred into congenic CD45.2^+^ recipients subsequently infected with LCMV-Arm (2 × 10^5^ plaque-forming units (PFU) intraperitoneally (IP) or LCMV-Cl13 (2 × 10^6^ PFU intravenously (IV)). For some experiments, in order to identify cells that had undergone their first division, CD8^+^ P14 T cells were first labeled with the proliferation dye carboxyfluorescein succinimidyl ester (CFSE) prior to transfer. For Division 1 analyses, 3 × 10^6^ cells were transferred, as previously described [24–26]; for Day 3 analyses, 1 × 10^6^ cells were transferred; for all other time point analyses, 1 × 10^4^ cells were transferred. Recipient mice were analyzed at 9 time points: days 2 (Division 1, “Div1”), 3, 5, 6, 7, 8, 22, 34, and 60 post-infection. Naïve CD8^+^ P14 T cells (CD44^lo^CD62L^hi^) were also included as a control. Donor CD8^+^CD45.1^+^ P14 T cells were FACS-purified at each time point and processed for scRNA-seq with the 10x Genomics Chromium platform (Figs 1A and S1).

To investigate the transcriptional differences between CD8^+^ T cells responding to acute versus chronic infection, we analyzed the data from all time points together and performed Uniform Manifold Approximation and Projection (UMAP) analyses. CD8^+^ T cells separated into 17 clusters (Fig 1B, left) on the basis of infection type (LCMV-Arm versus LCMV-Cl13) (Fig 1B, middle) and time points (Fig 1B, right). CD8^+^ T cells that had undergone their first division (second CFSE peak) in response to LCMV-Arm infection separated into two main clusters (Fig 1B, right), as previously observed [24]. Strikingly, CD8^+^ T cells that had undergone their first division in response to LCMV-Cl13 infection formed a single cluster that was distinct from the two LCMV-Arm Div1 clusters (Fig 1B, right).

Most clusters were made up of only cells responding to either LCMV-Arm or LCMV-Cl13 (Fig 1C). For example, Clusters 1, 3, 6, 9, 10, and 12 were mostly comprised of cells responding to LCMV-Arm, whereas Clusters 0, 7, 8, and 13 were primarily comprised of cells responding to LCMV-Cl13. The remainder of the clusters were comprised of mixtures of cells responding to either LCMV-Arm or LCMV-Cl13. Cells responding to LCMV-Arm harvested at different time points clustered distinctly, consistent with prior studies [24,27,28]. Cluster 0, which was comprised of cells responding to LCMV-Cl13 harvested at days 7 and 8 post-infection, contained progenitor exhausted and terminal exhausted cells (S2 Fig), consistent with a prior report [13]. Hierarchical clustering analyses grouped clusters exhibiting similar gene expression patterns (Fig 1D). Notably, grouping of clusters was driven by infection type, but also correlated with the time point after infection.

**Fig 2:**
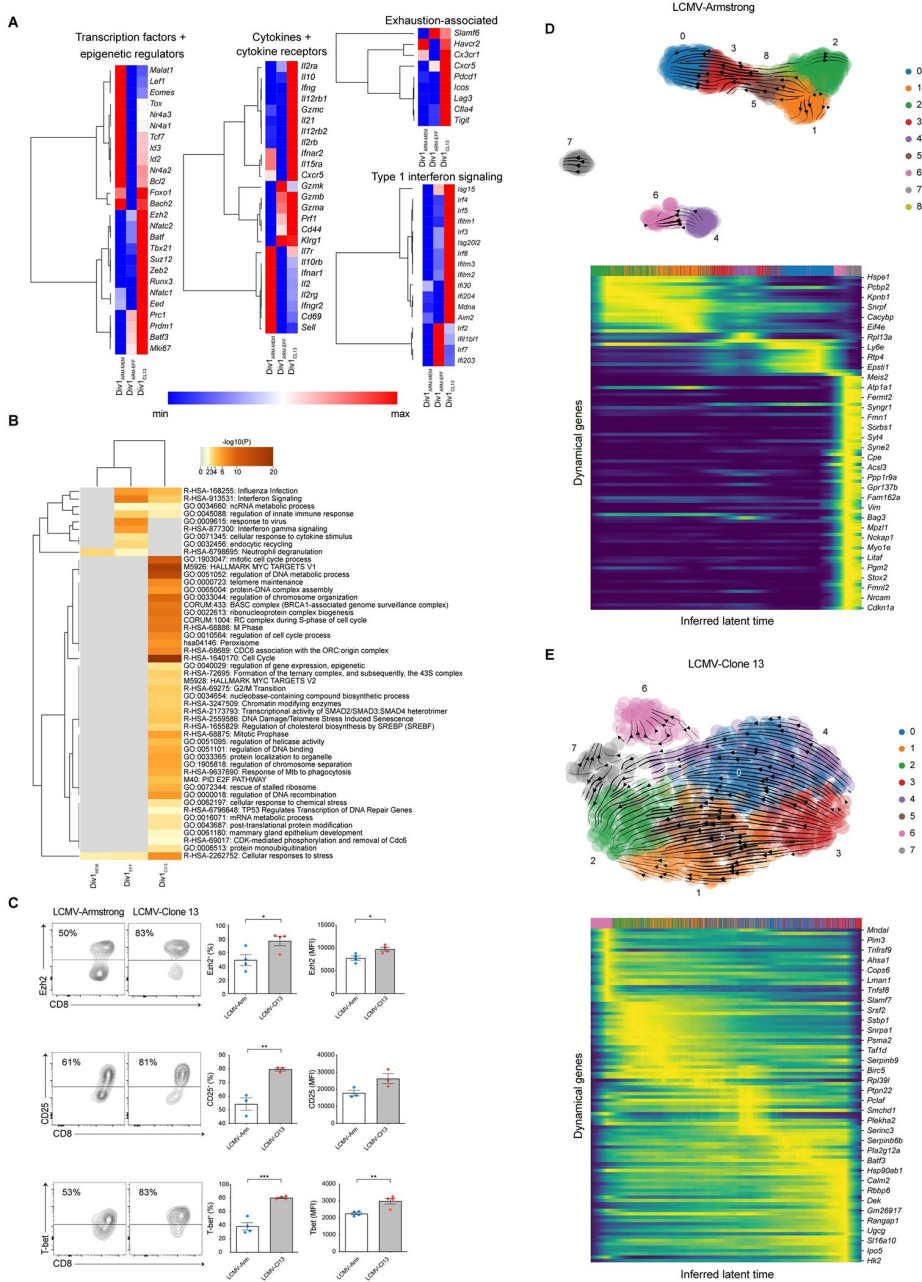
CD8^+^ T cells that have undergone their first division in response to LCMV-Arm vs. LCMV-Cl13 exhibit phenotypic and transcriptional heterogeneity. (**A**) Heatmaps representing relative gene expression of the three Division 1 clusters, Div1_ARM-EFF_, Div1_ARM-MEM_, and Div1_CL13_, divided by category; rows represent selected genes and columns represent each of three Division 1 clusters. (**B**) Enriched pathways among the three Division 1 clusters. (**C**) Representative flow cytometry plots (left) displaying expression of Ezh2, CD25 (IL-2Rα), and T-bet protein among gated Division 1 (second CFSE peak) P14 T cells. Bar graphs indicate the frequencies (middle) or MFI (right) of P14 T cells responding to LCMV-Arm (blue) or LCMV-Cl13 (red) expressing each molecule. Data are shown as mean ± SEM. (**D**, **E**) RNA velocities of Div1_ARM_ (D, top) or Div1_Cl13_ (E, top) subclusters derived from scVelo projected onto a UMAP-based embedding. Putative driver genes derived from scVelo that may regulate the CD8^+^ Division 1 T cell response to LMCV-Arm (D, bottom) vs. LCMV-Cl13 (E, bottom), represented as heatmaps. Individual lines above each heatmap represent single cells; colors correspond to subcluster identities among Div1_ARM_ (D, top) or Div1_Cl13_ (E, top) cells. *p < 0.05, **p < 0.01, ***p < 0.0001, ****p < 0.0001 (Student’s *t* test). Data are representative of 2 to 3 independent experiments. The raw data for the panels in this figure are located in S1 Data file. CFSE, carboxyfluorescein succinimidyl ester; LCMV-Arm, LCMV-Armstrong; LCMV-Cl13, LCMV-Clone 13; MFI, mean fluorescence intensity; UMAP, Uniform Manifold Approximation and Projection.

Next, to investigate the distinct clustering of CD8^+^ T cells that had undergone their first division in response to LCMV-Arm versus LCMV-Cl13, we analyzed the gene expression patterns of the three Div1 clusters. One of the two LCMV-Arm Div1 clusters expressed molecules associated with memory CD8^+^ T cells, whereas the other LCMV-Arm Div1 cluster expressed factors associated with terminal effector cell differentiation (Fig 2A and S1 and S2 Tables). We therefore annotated these two LCMV-Arm clusters as “Div1_ARM-MEM_” and “Div1_ARM-EFF_” because phenotypically similar clusters were previously shown to exhibit disparate tendencies to give rise to memory and effector CD8^+^ T cells [24]; the single LCMV-Cl13 cluster was annotated as “Div1_CL13_.” Pathway analyses of genes differentially expressed by the three Div1 clusters revealed an enrichment of genes related to proliferation, transcriptional, and epigenetic regulation, and chromatin modifying enzymes in the Div1_CL13_ cluster (Fig 2B). Focusing next on specific genes, we observed that transcription factors previously associated with memory CD8^+^ T cells, such as *Lef1*, *Eomes*, *Tcf7*, and *Id3*, were more highly expressed by Div1_ARM-MEM_ cells than by cells from either of the other two Div1 clusters (Fig 2A). By contrast, transcription factors, including *Irf4*, *Nfatc1*, and *Nfatc2*, which have been previously associated with exhaustion [9,14], were more highly expressed by Div1_Cl13_ cells. Furthermore, *Ezh2* and *Suz12*, which encode components of the PRC2 complex that mediates epigenetic silencing [23,24], were more highly expressed by Div1_Cl13_ cells. Lastly, Div1_Cl13_ cells expressed high levels of genes encoding molecules previously associated with exhaustion, including *Havcr2* (TIM3), *Lag3*, and *Pdcd1* (PD1), along with genes controlling responsiveness to cytokines including interleukin 2 (IL-2) and type I interferons (IFN-I). Flow cytometry experiments demonstrated that compared to Div1_ARM_ cells, higher proportions of Div1_Cl13_ cells expressed Ezh2, CD25 (*Il2ra*), and T-bet protein (Fig 2C).

To investigate whether the transcriptional heterogeneity observed in Div1 cells was accompanied by epigenetic heterogeneity, we performed the assay for transposase-accessible chromatin with high-throughput sequencing at the single-cell level (scATAC-seq) on Div1 cells. UMAP analyses using the scATAC-seq data revealed that Div1 cells separated into 4 clusters, three of which were derived from cells responding to LCMV-Cl13, and the other derived from cells responding to LCMV-Arm (S3 Fig). Intriguingly, additional analyses revealed that distinct sets of transcription factor binding motifs were preferentially enriched within differentially accessible chromatin peaks from the three Div1-Cl13 clusters. For example, motifs for BATF and AP-1 family transcription factors such as Fos and Jun were enriched in Div1-Cl13 Cluster 1; motifs for LEF and TCF transcription factors were enriched in Div1-Cl13 Cluster 3; and motifs for T-box transcription factors T-bet and Eomes were enriched in Div1-Cl13 Cluster 4 (S3 Fig). Taken together, these findings indicate that the differentiation paths of CD8^+^ T cells responding to acute versus chronic infection may diverge earlier than previously appreciated.

The lack of overlap between Div1_ARM_ and Div1_Cl13_ cells in UMAP analysis (Fig 1C) indicated that these cells did not to appear share a common differentiation pathway. To gain further insight into these distinct differentiation paths, we applied scVelo, a previously published framework to analyze transcriptional dynamics of splicing kinetics using a likelihood-based dynamical model [29,30]. We sought to identify putative “driver” genes that may regulate inferred RNA velocities in Div1_ARM_ versus Div1_Cl13_ subclusters. Putative driver genes identified by scVelo as influencing inferred velocities observed in Division 1 cells responding to LCMV-Cl13 were largely distinct from putative driver genes identified as regulating inferred velocities observed in Div1_ARM_ cells (Fig 2D and 2E and S3 Table), supporting the hypothesis that Div1_ARM_ and Div1_Cl13_ cells may undertake divergent differentiation paths. Although some identified putative driver genes may regulate paths of differentiation, others may simply be associated with the process; nonetheless, the genes identified by scVelo represent a starting point for further experimental study.

### Ezh2-mediated epigenetic silencing may regulate expression of exhaustion-associated molecules

**Fig 3:**
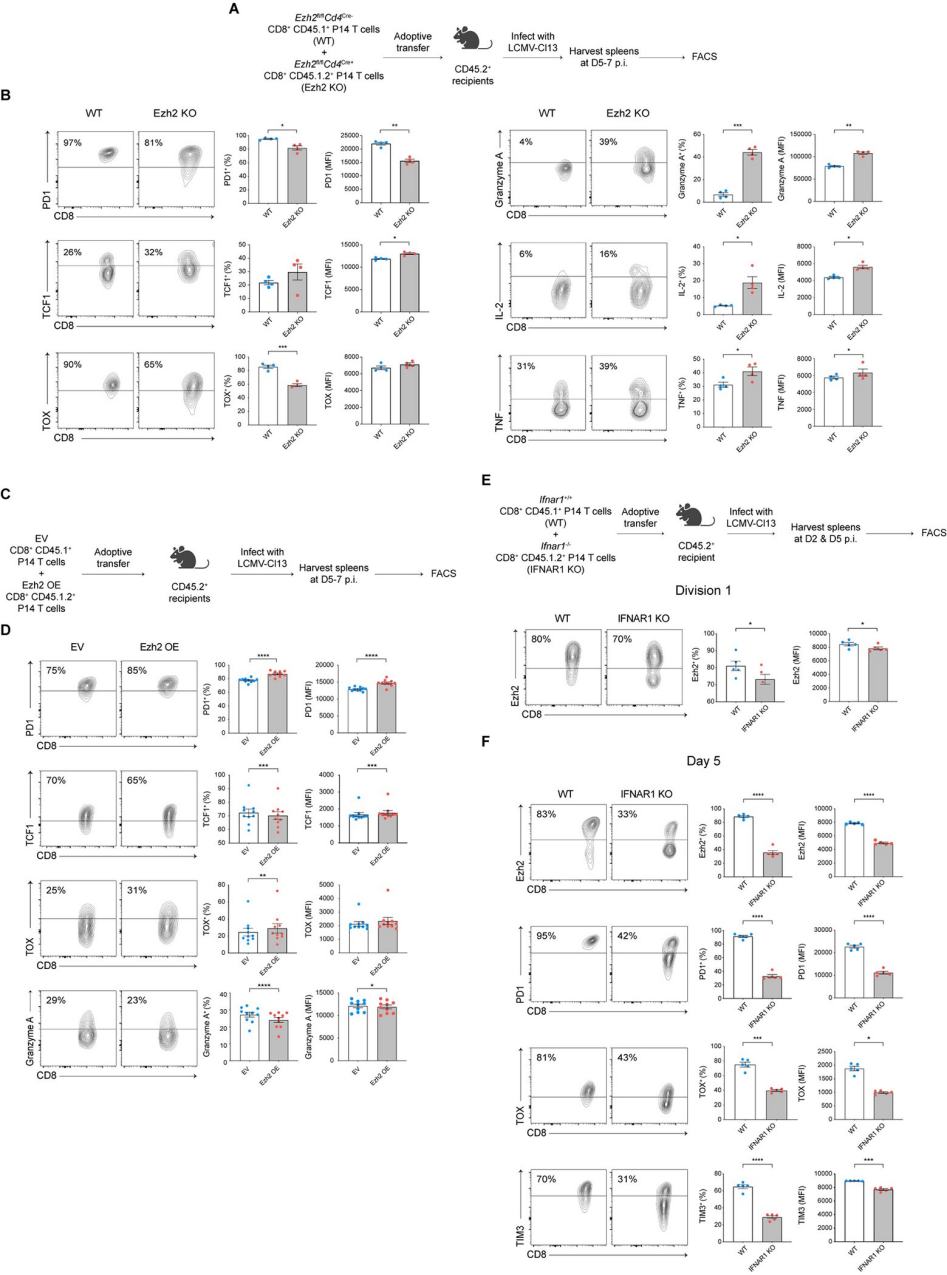
Ezh2-mediated epigenetic repression may partially influence exhaustion. (**A**) Experimental setup. Control CD45.1^+^ (wild-type, “WT”) and Ezh2-deficient CD45.1.2^+^(*Ezh2*^*fl*/*fl*^*Cd4*^*Cre+*^, “Ezh2 KO”) CD8^+^ P14 T cells were cotransferred into congenically distinct CD45.2^+^ recipient mice prior to infection with LCMV-Cl13; recipient mice were killed at 5–7 days post-infection and splenocytes analyzed by flow cytometry. (**B**) Representative flow cytometry plots (left) displaying expression of PD1, TCF1, TOX, Granzyme A, IL-2, or TNF protein among gated donor WT or Ezh2 KO P14 T cells. Bar graphs indicate the frequencies (middle) or MFI (right) of WT (blue) or Ezh2 KO (red) P14 T cells responding to LCMV-Cl13. (**C**) Experimental setup. CD8^+^ P14 T cells were transduced with an empty vector control (EV, CD45.1^+^) or Ezh2 overexpression (Ezh2-OE, CD45.1.2^+^) construct prior to adoptive cotransfer into CD45.2^+^ recipient mice prior to infection with LCMV-Cl13; recipient mice were killed at 5–7 days post-infection and splenocytes analyzed by flow cytometry. (**D**) Representative flow cytometry plots (left) displaying expression of PD1, TCF1, TOX, and Granzyme A among gated donor EV or Ezh2-OE P14 T cells. Bar graphs indicate the frequencies (middle) or MFI (right) of EV (blue) or Ezh2-OE (red) P14 T cells expressing each molecule. (**E**, **F**) Control CD45.1^+^ (wild-type, “WT”) and IFNAR1-deficient CD45.1.2^+^(*Ifnar1*^-/-^, “IFNAR1 KO”) CD8^+^ P14 T cells were labeled with CFSE (E only) and cotransferred into congenically distinct CD45.2^+^ recipient mice prior to infection with LCMV-Cl13; recipient mice were killed at 2 (E) or 5 days (F) post-infection and splenocytes analyzed by flow cytometry. Representative flow cytometry plots (left) displaying expression of Ezh2 protein among gated Division 1 (second CFSE peak) WT vs. IFNAR1 KO P14 T cells. Bar graphs indicate the frequencies (middle) or MFI (right) of WT (blue) or IFNAR1 KO (red) P14 T cells expressing each molecule. (**F**) Representative flow cytometry plots (left) displaying expression of Ezh2, PD1, TOX, and TIM3 protein among gated WT vs. IFNAR1 KO P14 T cells. Bar graphs indicate the frequencies (middle) or MFI (right) of WT (blue) or IFNAR1 KO (red) P14 T cells expressing each molecule. Data are shown as mean ± SEM. *p < 0.05, **p < 0.01, ***p < 0.0001, ****p < 0.0001 (paired *t* test). Data are representative of 2 to 3 independent experiments. The raw data for the panels in this figure are located in S1 Data file. Fig 3A, 3C and 3E created with BioRender.com. CFSE, carboxyfluorescein succinimidyl ester; EV, empty vector; IL-2, interleukin 2; LCMV-Cl13, LCMV-Clone 13; MFI, mean fluorescence intensity; TNF, tumor necrosis factor; WT, wild-type.

The observation that the gene encoding Ezh2, the enzymatic catalytic subunit of the repressive PRC2 complex, was up-regulated in Div1_Cl13_ cells (Fig 2A) raised the possibility that epigenetic silencing might be involved in regulating exhaustion. Consistent with this possibility, we observed a higher number of repressive H3K27me3 peaks, but not activating H3K4me3 peaks, in CD8^+^ T cells responding to LCMV-Cl13 compared to CD8^+^ T cells responding to LCMV-Arm at day 7 post-infection (S4 Fig). To experimentally test the hypothesis that epigenetic silencing might be involved in regulating exhaustion, we utilized *Ezh2*^*fl/fl*^*Cd4*^Cre+^(Ezh2-deficient) CD8^+^ P14 T cells, which, prior to activation, exhibited a naïve CD44^lo^CD62L^hi^ phenotype comparable to control counterparts [24], though it remains possible that Ezh2-deficient and control CD8^+^ P14 T cells may not be equivalent prior to adoptive transfer. Congenically distinct CD45.1^+^ control and CD45.1.2^+^ Ezh2-deficient CD8^+^ P14 T cells were adoptively cotransferred at a 1:1 ratio into CD45.2^+^ recipients subsequently infected with LCMV-Cl13 and analyzed by flow cytometry at 5 days post-infection (Fig 3A). Compared to control cells, Ezh2-deficient T cells exhibited reduced expression of the exhaustion-associated molecules PD1 and TOX, along with increased expression of TCF1 (Fig 3B, left). Furthermore, Ezh2-deficient T cells exhibited increased expression of Granzyme A, IL-2, and TNF (Fig 3B, right). Similar results were observed with Ezh2-heterozygous (*Ezh2*^*fl/wt*^*Cd4*^Cre+^) CD8^+^ P14 T cells (S5 Fig).

In parallel, we asked whether forced expression of Ezh2 might result in increased expression of exhaustion-associated molecules. Congenically distinct CD8^+^ P14 T cells were transduced with empty vector (EV, CD45.1^+^) or Ezh2 retroviral constructs (Ezh2 OE, CD45.1.2^+^), mixed at a 1:1 ratio, and adoptively transferred into CD45.2^+^ recipients prior to infection with LCMV-Cl13 (Fig 3C). Compared to control cells, Ezh2 OE CD8^+^ P14 T cells exhibited increased expression of PD1 and TOX, along with reduced expression of TCF1 and Granzyme A (Fig 3D). Taken together, these findings suggest a possible partial role for Ezh2-mediated epigenetic silencing in regulating exhaustion.

Lastly, we asked what factors might mediate Ezh2 up-regulation during the early CD8^+^ T cell response to LCMV-Cl13. The observation that genes associated with IFN-I signaling were up-regulated in Div1_Cl13_ cells compared to Div1_ARM_ cells (Fig 2A) raised the possibility that IFN-I signaling might regulate Ezh2 expression. We therefore tested whether deletion of IFNAR1, the receptor for IFN-I, in CD8^+^ T cells might affect Ezh2 expression in Div1 cells. Congenically distinct control (CD45.1^+^) or *Ifnar1*^-/-^ (CD45.1.2^+^, IFNAR1-deficient) CD8^+^ P14 T cells were labeled with CFSE, mixed at a 1:1 ratio, and adoptively transferred into CD45.2^+^ recipients prior to infection with LCMV-Cl13 and cells that had undergone their first division (second CFSE peak) were analyzed. Compared to control cells, IFNAR1-deficient Division 1 cells exhibited reduced expression of Ezh2 (Fig 3E), indicating that IFN-I signaling may mediate up-regulation of Ezh2 in CD8^+^ T cells responding to LCMV-Cl13. Furthermore, at day 5 post-infection, IFNAR1-deficient CD8^+^ P14 T cells continued to exhibit reduced Ezh2 expression, along with reduced expression of exhaustion-associated molecules, including PD1, TIM3, and TOX, compared to control cells (Fig 3F). Similar results were observed with antibody-mediated blocking of IFNAR1 in CD8^+^ P14 T cells (S6 Fig). Taken together, these results suggest that IFN-I signaling may play a role in inducing up-regulation of Ezh2 in Div1-Cl13 cells, which, in turn, may mediate epigenetic repression and contribute, in part, to promoting exhaustion.

## Discussion

Although substantial progress has been made in elucidating the transcriptional and epigenetic regulation of exhaustion, the precise sequence of events controlling the formation of T_EX_ cells remains incompletely understood. In particular, from what precursor cells are T_EX_ cells derived? It was initially thought that T_EX_ cells were derived from terminally differentiated effector cells. However, lineage tracing experiments demonstrated that CD127^hi^KLRG1^lo^ memory precursor T cells, but not KLRG1^hi^ effector T cells, can give rise to T_EX_ cells [2]. Another study using a combination of scRNA-seq, lineage tracing, and genetic perturbations defined some of the early features of T_EX_ cell formation [21]. TCF1 was shown to govern early events by antagonizing genes that promote terminal effector differentiation while positively regulating Eomes and c-Myb. These actions orchestrated a divergence of the T_EX_ cell versus terminal effector cell differentiation pathways that was evident by day 8 following infection with LCMV-Cl13. Lastly, Utzschneider and colleagues demonstrated that high antigen load promoted the differentiation of TCF1^+^ precursor T cells, which acquired hallmarks of exhaustion within days of infection, whereas early effector T cells resembled polyfunctional T cells that respond to acute infection [31]. These findings suggest a model in which T cell exhaustion is first established in TCF1^+^ precursor T cells and subsequently propagated to the pool of antigen-specific T cells.

A prior scRNA-seq study identified a transcriptional divergence between CD8^+^ T cells responding to LCMV-Arm versus LCMV-Cl13 occurring after day 4.5 but before day 7 post-infection [13]. By contrast, our data raise the possibility that CD8^+^ T cells that have undergone their first division in response to LCMV-Cl13 may already be proceeding along a differentiation path that is transcriptionally and epigenetically distinct from those responding to LCMV-Arm. Strikingly, Div1_ARM_ cells exhibited transcriptional but not epigenetic heterogeneity, whereas Div1_CL13_ cells exhibited epigenetic but not transcriptional heterogeneity. The functional importance of these transcriptional and epigenetic disparities, as well as possible relationships between putative epigenetic Div1_CL13_ states and previously described TCF1^+^ precursor and early effector T cells [31] remain to be determined. Furthermore, in light of a prior study showing that restimulated CD8^+^ T cells FACS-isolated 30 days after LCMV-Cl13 infection exhibited a reduced capacity to undergo asymmetric cell division [32], which has been shown to result in differentially fated progeny during acute infection [26,33], it is intriguing to speculate that formation of the transcriptionally homogenous Div1_CL13_ population may result, in part, from an impaired capability to undergo asymmetric division. Future studies will investigate this possibility as well as the transcriptional and epigenetic changes following the first CD8^+^ T cell division in response to LCMV-Cl13.

The transcriptional differences observed suggest that there may be early differences in signaling between cells responding to LCMV-Arm versus LCMV-Cl13. What factors might account for these differences? One possibility is that viral antigen load may be substantially higher at early time points in LCMV-Cl13 compared to LCMV-Arm infection, potentially leading to increased TCR signaling. In this study, we administered LCMV-Arm at 2 × 10^5^ PFU IP and LCMV-Cl13 at 2 × 10^6^ PFU IV as these infection doses and routes have been routinely used to model acute and chronic infections [34,35], respectively, but they may have resulted in differences in viral antigen loads. Moreover, it should be noted that even at identical doses and routes of infection, infection with LCMV-Cl13 resulted in higher proportions of infected plasmacytoid dendritic cells (pDCs) and higher levels of serum IFNα and IFNβ at 24 hours post-infection compared to infection with LCMV-Arm [36,37].

Alternatively, it has been reported that LCMV-Arm and LCMV-Cl13 may preferentially localize to different regions of the spleen. LCMV binds to the receptor alpha-dystroglycan (αDG) expressed on splenic dendritic cells (DCs) [38,39]. Strains and variants that bind αDG with high affinity, such as LCMV-Cl13, are associated with viral replication in the white pulp, show preferential replication in splenic DCs, and establish a persistent infection [40]. In contrast, viral strains and variants that bind with low affinity to αDG, such as LCMV-Arm, are associated with viral replication in the red pulp, display minimal replication in splenic DCs, and generate a robust CD8^+^ T cell response that clears the infection. Taken together, differences in viral antigen load, cell tropism, cytokine milieu, and/or anatomic localization of LCMV-Arm versus LCMV-Cl13 in the spleen could influence the acquisition of distinct transcriptional and epigenetic profiles observed in Division 1 CD8^+^ T cells.

T_EX_ cells differ from effector and memory T cells by approximately 6,000 open chromatin regions [41–44]. This epigenetic divergence is evident by day 5 post-infection and becomes progressively more widespread and permanent with time, resulting in durable epigenetic “scars” [34]. Our observation that CD8^+^ T cells that had undergone their first division in response to LCMV-Cl13 exhibited heterogeneity on the basis of their chromatin accessibility patterns suggests the possibility that epigenetic changes may begin to occur earlier than previously appreciated. With regard to the specific epigenetic changes that regulate exhaustion, several prior reports have suggested a possible role for epigenetic silencing, analogous to its role in terminal effector cell differentiation, in which Ezh2 mediates repression of genes associated with memory cell differentiation [23,24]. For example, application of an integrative network modeling approach suggested that epigenetic silencing mediated by Ezh2 may play a role in regulating exhaustion [45]. Furthermore, the microRNA miR-155 increased CD8^+^ T cell antitumor function by restraining T cell senescence and functional exhaustion through PRC2-mediated epigenetic silencing of transcription factors driving terminal differentiation and exhaustion [46]. Consistent with these results, we observed that at day 7 post-infection, CD8^+^ T cells responding to LCMV-Cl13 exhibited increased H3K27me3 repressive marks compared to CD8^+^ T cells responding to LCMV-Arm. Furthermore, Ezh2 deficiency resulted in reduced expression of exhaustion-associated molecules by CD8^+^ T cells responding to LCMV-Cl13, whereas forced Ezh2 expression resulted in increased expression of exhaustion-associated molecules, supporting the hypothesis that epigenetic silencing may play a partial role in the molecular regulation of exhaustion. In addition, our data suggest that responsiveness to IFN-I signaling may be involved in the initial up-regulation of Ezh2 in CD8^+^ T cells responding to LCMV-Cl13. However, since Ezh2 also plays a role in CD8^+^ T cell differentiation in response to acute infection [23,24], it is likely that the specific cellular and infectious contexts, rather than simply the level of Ezh2 expression, determine the outcome of terminal effector differentiation, memory cell generation, or exhaustion. Future studies will focus on identifying the cellular sources of IFN-I that induce Ezh2 expression as well as elucidating the timing and specific mechanisms by which epigenetic silencing may regulate T cell exhaustion.

## Materials and methods

### Ethics statement

This study involved vertebrate animals. All mice were housed under specific pathogen-free conditions in an American Association of Laboratory Animal Care-approved facility at the University of California San Diego (UCSD), and all procedures were approved by the UCSD Institutional Animal Care and Use Committee under protocol number S09264. C57BL6/J, P14 TCR transgenic (CD45.1, CD45.2 or CD45.1.2), *Ezh2*^*fl/fl*^, *Ifnar1*^-/-^, and *Cd4*^*Cre*^ mice were purchased from Jackson Laboratories or bred at UCSD. All mice were used from 6 to 9 weeks of age, male mice were used as recipients, and male or female mice were used as donors in adoptive transfer experiments.

### Antibodies, flow cytometry, and cell sorting

Cells were stained for 15 minutes on ice with the following antibodies: Vβ8.1/8.2 (MR5-2), CD8α (53–6.7), CD45.1 (A20), CD45.2 (104), Ezh2 (11/Ezh2), H3K27me3 (C36B11), PD1 (29F.1A12), TIM3 (RMT3-23), CD25 (PC61), CD44 (IM7), TOX (REA473), SLAMF6 (330-AJ), EOMES (Dan11mag), TCF1 (C63D9), and Granzyme A (3G8.5). Antibodies targeting TCF1 and H3K27me3 were purchased from Cell Signaling Technology, anti-TOX mAbs were purchased from Miltenyi Biotec, anti-Granzyme A mAbs were purchased from Thermo Fisher Scientific, and the remainder were purchased from Biolegend. Samples were then stained with Fixable Viability Dye eFluor780 (Thermo Fisher Scientific), for 15 minutes on ice. For all intracellular stains, cells were fixed in either 2% paraformaldehyde, or fixed and permeabilized with the FoxP3/Transcription Factor Staining Buffer Kit (Thermo Fisher Scientific). For analysis, all samples were run on an Accuri C6, LSRFortessa X-20 (BD Biosciences) or Novocyte (Agilent Biosciences). For sorting, all samples were run on an Influx, FACSAria Fusion or FACSAria2 (BD Biosciences). BD FACS DIVA (BD Biosciences) or NovoExpress (Agilent Biosciences) software was used for data collection, and FlowJo software (BD Biosciences) was used for analysis of flow cytometry data.

### In vivo mouse experiments

Spleens were dissected from naïve donor CD45.1^+^ or CD45.1.2^+^ P14 mice and made into a single-cell suspension. Cells were centrifuged and subjected to red blood cell lysis, washed several times, and filtered through a 70-μm cell strainer. No collagenase was used in the processing of spleens. Cells were stained with anti-Vβ8.1/8.2, anti-CD8α, and anti-CD45.1 mAbs. As the majority of P14 cells (approximately 84% to 90%) typically exhibited a naïve CD44^lo^CD62L^hi^ phenotype (S1A Fig), we did not specifically FACS-purify donor P14 cells prior to adoptive transfer, similar to other prior studies [24,27,47,48]. P14 cells were then adoptively transferred into congenically distinct wild-type recipients before infection with either 2 × 10^5^ PFU of LCMV-Arm or 2 × 10^6^ PFU LCMV-Cl13. LCMV-Arm was injected IP, and LCMV-Cl13 was injected IV. To conduct the genetic deficiency experiments, congenically distinct splenocytes from either *Ezh2*^*fl/fl*^*Cd4*^*Cre+*^ and *Ezh2*^*fl/fl*^*Cd4*^*Cre*-^ P14 mice; or *Ezh2*^*fl/wt*^*Cd4*^*Cre+*^ and *Ezh2*^*fl/wt*^*Cd4*^*Cre*-^ P14 mice; or *Ifnar1*^-/-^ and *Ifnar1*^+/+^ P14 mice were harvested, stained, and counted as above and adoptively transferred at a 1:1 ratio into congenically distinct hosts. These mice were then infected intravenously with 2 × 10^6^ PFU LCMV-Cl13. For Division 1 analyses, 3 × 10^6^ cells were transferred; for Day 3 analyses, 1 × 10^6^ cells were transferred; for all other time point analyses, 1 × 10^4^ cells were transferred. For adoptive cotransfer experiments analyzed at all other time points post-infection, a total of 2 × 10^4^ cells were transferred. For analysis of Division 1 cells, we transferred 3 × 10^6^ cells because this high cell number is required in order to be able to detect sufficient numbers of Division 1 cells for FACS analysis or to perform FACS-isolation for scRNA-seq. For this reason, all published papers to date analyzing Division 1 cells responding to infection in vivo have used similarly high numbers of cells for adoptive transfer [24–26,33,49–52]. However, it should be noted that starting frequencies of donor P14 cells have a major impact on the pace of T cell differentiation following infection and could impact kinetics of viral clearance and severity of exhaustion. Thus, the different numbers of donor P14 cells transferred is a confounding factor.

### 10x Genomics library preparation and sequencing

P14 T cells (CD8^+^CD45.1^+^) were sorted from the spleen and resuspended in phosphate-buffered saline (PBS) + 0.04% (w/v) bovine serum albumin. About 10,000 cells per sample were loaded into Single Cell A chips or Single Cell G chips (10x Genomics) and partitioned into Gel Bead In-Emulsions in a Chromium Controller (10x Genomics). Single-Cell RNA libraries were prepared according to the 10x Genomics Chromium Single-Cell 3′ Reagent Kits v2 User Guide or Next GEM Single Cell 3′ Reagent kits v3.1 User Guide and sequenced on a HiSeq 4000 (Illumina).

### Ezh2 overexpression experiments

The MSCV-mouse-Ezh2-IRES-GFP (Ezh2 overexpression, Ezh2-OE) vector was a gift from Martine Roussel (Addgene plasmid #107146; http://n2t.net/addgene:107146; RRID:Addgene 107146). To generate retroviral particles, Platinum-E (Plat-E) cells were plated in 10 cm plates 1 day prior to transfection and transfected with 10 μg of the Ezh2-OE or empty vector and 5 μg of pCL-Eco using TransIT-LTI (Mirus). The supernatant was collected at 48 and 72 hours post-transfection and stored at −80°C. For transduction, spleens and lymph nodes were harvested from naïve CD45.1^+^ and CD45.1.2^+^ P14 mice and whole splenocytes were activated in vitro with LCMV GP33_33–41_ peptide for 1 hour at 37°C. Around 1 × 10^6^ activated splenocytes were plated in a 48-well plate and incubated for 36 hours at 37°C. Retroviral supernatant was added to the plated cells, supplemented with polybrene (8 μg/mL, Millipore), and centrifuged for 90 minutes at 900 rcf at room temperature. Retroviral supernatant was replaced with culture medium (Isocove’s modified Dulbecco’s medium + 10% fetal bovine serum (v/v) + 2 mM glutamine + penicillin (100 U/mL) + streptomycin (100 μg/ml) + 55 mM β-mercaptoethanol), and cells were rested for 2 hours at 37°C. Cells were washed 3 times with PBS and counted. Based on a previous test of transduction efficiency, a 1:1 ratio of P14 cells transduced with EV and Ezh2-OE retroviruses (total of 2 × 10^4^ P14 cells) were adoptively transferred into a congenically distinct host. One hour later, recipient mice were infected with 2 × 10^6^ PFU IV of LCMV-Cl13. Five to seven days later, spleens were harvested and analyzed by flow cytometry.

### Single-cell RNA-seq data analysis

The scRNA-seq data were aligned and quantified using the Cell Ranger Single-Cell Software Suite against the GRCm38 (mm10) mouse reference genome. The preliminary filtered data generated from Cell Ranger were used for the downstream analysis. Further quality control was applied to cells based on two metrics, including the number of detected genes and proportion of mitochondrial gene count per cell. Specifically, cells with less than 200 detected genes were excluded, as well as cells with more than 30% mitochondrial gene count. Genes that were expressed in less than 3 cells in the dataset were also removed. After quality control, we normalized the sequencing depth for each cell by applying the *normalize_total* function in Scanpy [53] to the raw counts. The logarithmized normalized count matrix was then used for the downstream analysis. The normalized and logarithmized single-cell data were processed for dimension reduction and unsupervised clustering following the workflow in Scanpy [53]. In brief, a principal component analysis (PCA) matrix was first calculated to reveal the main axes of variation and denoise the data by using *scanpy*.*tl*.*pca* function with default parameters. For visualization, the dimensionality of each dataset was further reduced using UMAP implemented in the *scanpy*.*tl*.*umap* function with default parameters. We used Leiden, an unsupervised graph-based clustering algorithm, to cluster single-cells by their expression profiles, with *sc*.*tl*.*leiden* function and default settings. The differentially expressed genes were identified by using the *scanpy*.*tl*.*rank_genes_groups* function with default parameters.

### RNA velocity analysis

The aligned single-cell reads (in BAM files) from Cell Ranger software were first counted for spliced and unspliced mRNAs using the velocyto package [30]. The velocity estimation and visualization of the samples were then obtained with the scVelo package [29]. We first computed the first- and second-order moments for velocity estimation using the *scvelo*.*pp*.*filter_and_normalize* and *scvelo*.*pp*.*moments* functions with default settings. RNA velocity was then estimated with the generalized dynamical model in scVelo using *scv*.*tl*.*recover_dynamics* and *scvelo*.*tl*.*velocity*. We used the *scv*.*tl*.*velocity_graph* function to project the velocities onto a lower-dimensional embedding (UMAP) by translating them into likely cell transitions and to calculate the probabilities of one cell transitioning into another cell. *scvelo*.*pl*.*velocity_embedding_stream* was used to visualize the velocities. The latent time status for each cell was also estimated from the velocities using the dynamical model with *scvelo*.*tl*.*latent_time* function while the driver genes for the dynamics were also predicted. The *scv*.*pl*.*scatter* function was used to visualize the latent time status and driver genes. It should be noted that the putative driver genes identified by scVelo are drivers of the inferred velocities, not necessarily drivers of a cellular program. However, some of these driver genes may actually be bona fide drivers of a transcriptional program, while other genes may simply be associated with a process but not drive it. The putative driver genes identified by scVelo are intended to be a starting point for further experimental study. The lists provided in S3 Table are the top 100 driver genes identified by scVelo, whereas the genes shown in the Fig 2D and 2E heatmaps are 34 of these 100 genes presented on the heatmap using the default settings for scVelo. scVelo selects every third gene of the top 100 genes (which are ranked in order of inferred latent time), thereby resulting in 34 genes selected for inclusion in each heatmap.

### Single-cell ATAC-seq analysis

The Cellranger ATAC pipeline (1.2.0) [54] was used to preprocess the sequencing data. Firstly, we started from fastq files and the reads were mapped to mm10 genome using cellranger-atac count program. Peaks were also identified within each sample individually. We next pooled the returned results from all samples to produce a single peak-barcode matrix using cellranger-atac aggr with option—normalize = signal. This enables the direct comparison between groups (i.e., LCMV-Arm versus LCMV-Cl13) in the downstream analysis. The returned aggregated files were loaded into Signac (1.5.0) [55], an R (4.0.2) package, for downstream analysis using the standard Signac/Seurat pipeline. With Signac, QC metrics were first calculated for each cell, which include the total number of fragments in peaks, fraction of all fragments that fall within ATAC-seq peaks, nucleosome signal strength, and the ratio of reads in genomic blacklist regions provided by ENCODE project [56]. Outlier cells in the QC metric categories were removed per Signac’s standard processing guidelines. Differentially accessible regions were identified by *FindMarkers* function, and each peak was also annotated by its closest gene using *ClosestFeature*.

Latent semantic indexing (LSI), a form of dimensional reduction, was performed using Signac’s “*RunTFIDF*” and “*RunSVD*” functions. LSI dimensions that were highly correlated with read depth were identified using Signac’s “DepthCor” and were not used in downstream analysis. The UMAP hyperparameters were varied to produce consistent object shapes (using R). Once hyperparameters were chosen, we ran Signac/Seurat’s “*RunUMAP*” function on the LSI dimensions chosen earlier to compute the UMAP embedding. Signac/Seurat’s “*FindNeighbors*” function was run using the same LSI dimensions as UMAP to compute the nearest neighbors graph. Signac/Seurat’s “*FindClusters*” was then run to identify the clusters of the cells with resolution set to 0.2. Additionally, the read density was visualized in Integrated Genome Browser [57] using the bigwig files generated in the *cellranger-atac* count step. The heatmap and density plots were generated using the same bigwig files and the bed files of differentially accessible peaks with the *computeMatrix* and *plotHeatmap* functions of DeepTools software.

### H3K27me3 and H3K4me3 deposition analysis

CD8^+^CD45.1^+^ P14 cells were adoptively transferred into CD45.2^+^ recipients subsequently infected with LCMV-Arm or LCMV-Cl13 as described above. At day 7 post-infection, mice were killed and donor CD8^+^CD45.1^+^ P14 cells were FACS-isolated, stained with anti-H3K4me3, anti-H3K27me3, or isotype IgG control mAbs (Cell Signaling), and processed for the CUT&RUN Assay Kit (Cell Signaling). CUT&RUN libraries were sequenced from both ends using an Illumina HiSeq 4000 to a total read length of 101 bp from each end. The reads were firstly trimmed with trimmomatic v0.36 to remove the sequencing adapters and then aligned against the mouse genome (GRCm38) using Bowtie2 [58] with parameters set as—local—very-sensitive-local—no-unal—no-mixed—no-discordant—phred33 -I 10 -X 700. Spike-in normalization is used for calibrating the epitope abundance between experiments as described [59]. In the spike-in normalization, the trimmed reads were also aligned against yeast genome (sacCer3) with Bowtie2 with two more parameters—no-overlap and—no-dovetail, to avoid possible cross-mapping of the experimental genome to that of the carry-over yeast DNA, which is used for calibration. The genomic coverage was then normalized by applying the scaling factor that is calculated from the number of mapped reads to mouse genome and yeast genome [59]. MACS2 [60] was used for peak calling analysis: (1) we first used the Callpeak program to obtain the peaks for each sample based on the spike-in normalized alignment files; (2) we next used the bdgcmp program to compare H3K4me3 or H3K27me3 samples against their corresponding IgG samples, which generated the relative binding signals from read signals (i.e., the fold-enrichment of H3K4me3 or H3K27me3 against IgG samples) for each peak region found in (1). The returned BedGraph files in (2) were visualized in Integrated Genome Browser [57]. The heatmap and density plots were generated using the relative binding signals with the computeMatrix and plotHeatmap functions of DeepTools software. Motif analysis on the peak regions were implemented using findMotifsGenome.pl program in HOMER software.

### Functional enrichment analysis

Pathway analyses were implemented with PANTHER using Fisher’s exact test and the default settings [61] or Metascape [62].

## Supporting information

S1 Table Average gene expression for all samples and clusters shown in Fig 1B. (XLSX)

S2 Table Differentially expressed genes for the three Division 1 clusters identified in Fig 1B. (XLSX)

S3 Table Top putative driver genes for Div1_ARM_ vs. Div1_Cl13_ velocities identified by scVelo in Fig 2D and 2E. (XLSX)

S1 Data Raw data related to each individual figure. (XLSX)

S1 Fig Gating strategy for FACS-purification of Division 1 CD8^+^ P14 T cells. (**A**) Representative flow cytometry plot showing CD62L and CD44 expression of wild-type CD8^+^ P14 T cells. (**B**) Gating strategy for FACS-purification of Division 1 CD8^+^ P14 T cells for subsequent downstream analyses.
(TIF)

S2 Fig UMAP reclustering analyses of Cluster 0. (**A**) Cells from Cluster 0 (see Fig 1B), which was comprised of Day 7 and Day 8 cells responding to LCMV-Cl13, were reclustered and results are presented as a new UMAP. Reclustering of Cluster 0 resulted in two subclusters (0 and 1). (**B**) Expression of *Tcf7*, *Cxcr5*, *Slamf6*, and *Havcr2* by subclusters 0 and 1, represented as violin plots. Each violin represents the probability density at each value; each dot represents one cell. LCMV-Cl13, LCMV-Clone 13; UMAP, Uniform Manifold Approximation and Projection.
(TIF)

S3 Fig scATAC-seq analyses reveal epigenetic heterogeneity among CD8^+^ T cells that have undergone their first division in response to LCMV-Arm vs. LCMV-Cl13. CD8^+^CD45.1^+^ P14 T cells were CSFE-labeled prior to adoptive transfer into separate CD45.2^+^ recipient mice that were infected with LCMV-Arm or LCMV-Cl13. Recipient mice were killed at 2 days post-infection and Division 1 (second CFSE peak) P14 T cells were FACS-isolated; nuclei were extracted and processed for scATAC-seq using the 10x Genomics pipeline. (**A**) UMAP clustering of all CD8^+^ cells on the basis of scATAC-seq data colored by infection type (left) or cluster identity (right) is shown. (**B**-**D**) Selected examples of transcription factor motifs preferentially enriched in accessible chromatin regions from each of the three Div1_CL13_ clusters. CFSE, carboxyfluorescein succinimidyl ester; LCMV-Arm, LCMV-Armstrong; LCMV-Cl13, LCMV-Clone 13; scATAC-seq, single-cell assay for transposase-accessible chromatin using sequencing; UMAP, Uniform Manifold Approximation and Projection.
(TIF)

S4 Fig Increased H3K27me3 deposition in CD8^+^ T cells responding to LCMV-Cl13 compared to those responding to LCMV-Arm. (**A**) Experimental setup. (**B**, **C**) Venn diagram analysis of shared and differential H3K27me3 (B) or H3K4me3 (C) peaks identified in accessible chromatin regions from CD8^+^ T cells responding to LCMV-Arm vs. LCMV-Cl13. The raw data for the panels in this figure are located in S1 Data file. S4A Fig created with BioRender.com. LCMV-Arm, LCMV-Armstrong; LCMV-Cl13, LCMV-Clone 13.
(TIF)

S5 Fig Ezh2-mediated epigenetic silencing may influence expression of exhaustion-associated molecules. (**A**) Experimental setup. Control CD45.1^+^ (wild-type, WT) and Ezh2-heterozygous CD45.1.2^*+*^*(Ezh2*^*fl/wt*^*Cd4*^*Cre+*^, Ezh2 HET) CD8^+^ P14 T cells were cotransferred into congenically distinct CD45.2^+^ recipient mice prior to infection with LCMV-Cl13; recipient mice were killed at 5–7 days post-infection and splenocytes analyzed by flow cytometry. (**B**) Representative flow cytometry plots (left) displaying expression of PD1, TCF1, TOX, or Granzyme A protein among gated donor WT or Ezh2 HET P14 T cells. Bar graphs indicate the frequencies (middle) or MFI (right) of WT (blue) or Ezh2 HET (red) P14 T cells responding to LCMV-Cl13. Data are shown as mean ± SEM. **p < 0.01, ***p < 0.0001 (paired *t* test). Data are representative of 2 to 3 independent experiments. The raw data for the panels in this figure are located in S1 Data file. S5A Fig created with BioRender.com. LCMV-Cl13, LCMV-Clone 13; MFI, mean fluorescence intensity; WT, wild-type.
(TIF)

S6 Fig IFN-I signaling may induce Ezh2 expression in CD8^+^ T cells that have undergone their first division. (**A**) Experimental setup. CD45.1^+^ P14 T cells were transferred into separate CD45.2^+^ recipient mice prior to infection with LCMV-Cl13. For analysis of Division 1 cells, P14 cells were labeled with CFSE prior to transfer. Mice were treated with control isotype mAbs or anti-IFNAR1 blocking mAbs on the day of transfer (day -1) and day of infection (day 0). For analysis performed at day 5 post-infection, antibodies were also administered on days 2 and 4 post-infection. Mice were killed on day 2 or 5 post-infection for flow cytometry analysis. (**B**) Representative flow cytometry plots (left) displaying expression of Ezh2 protein among gated Division 1 (second CFSE peak) isotype- vs. anti-IFNAR1-treated P14 T cells. Bar graphs indicate the frequencies (middle) or mean fluorescence intensity (MFI, right) of isotype- (blue) or anti-IFNAR1-treated (red) P14 T cells expressing Ezh2. (**C**) Representative flow cytometry plots (left) displaying expression of Ezh2, PD1, TOX, and TIM3 protein among isotype- vs. anti-IFNAR1-treated P14 T cells. Bar graphs indicate the frequencies (middle) or MFI (right) of isotype- (blue) or anti-IFNAR1-treated (red) P14 T cells expressing each molecule. Data are shown as mean ± SEM. *p < 0.05, **p < 0.01, ***p < 0.0001 (Student’s *t* test). Data are representative of 2 to 3 independent experiments. The raw data for the panels in this figure are located in S1 Data file. S6A Fig created with BioRender.com. CFSE, carboxyfluorescein succinimidyl ester; IFN-I, type I interferon; LCMV-Cl13, LCMV-Clone 13; MFI, mean fluorescence intensity.
(TIF)

## Abbreviations

αDG: alpha-dystroglycan
CFSE: carboxyfluorescein succinimidyl ester
DC: dendritic cell
EV: empty vector
IFN-I: type I interferon
IFNγ: interferon gamma
IL-2: interleukin 2
IP: intraperitoneally
IV: intravenously
LCMV: lymphocytic choriomeningitis virus
LCMV-Arm: LCMV-Armstrong
LCMV-Cl13: LCMV-Clone 13
LSI: latent semantic indexing
PCA: principal component analysis
pDC: plasmacytoid dendritic cell
PFU: plaque-forming unit
PRC2: Polycomb Repressive Complex 2
scRNA-seq: single-cell RNA-sequencing
scATAC-seq: assay for transposase-accessible chromatin with high-throughput sequencing at the single-cell level
TCR: T cell receptor
TNF: tumor necrosis factor
UMAP: Uniform Manifold Approximation and Projection
