# Supplementary figures and images for: Early transcriptional and epigenetic divergence of CD8+ T cells responding to acute versus chronic infection
Source: PLoS Biol. 2023 Jan 30;21(1):e3001983. doi: 10.1371/journal.pbio.3001983 (PMC9886247; doi:10.1371/journal.pbio.3001983)

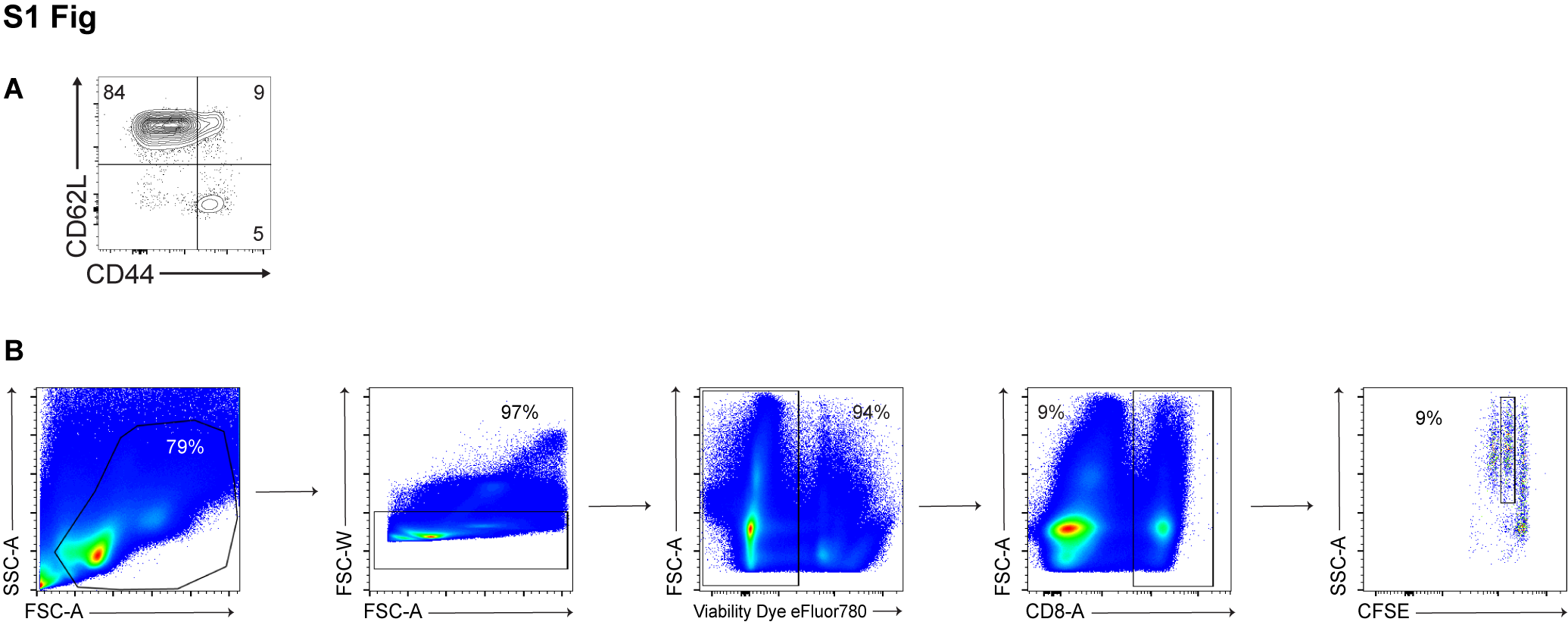

Supplement: S1 Fig — (A) Representative flow cytometry plot showing CD62L and CD44 expression of wild-type CD8+ P14 T cells. (B) Gating strategy for FACS-purification of Division 1 CD8+ P14 T cells for subsequent downstream analyses. (TIF) [file pbio.3001983.s005.tif]

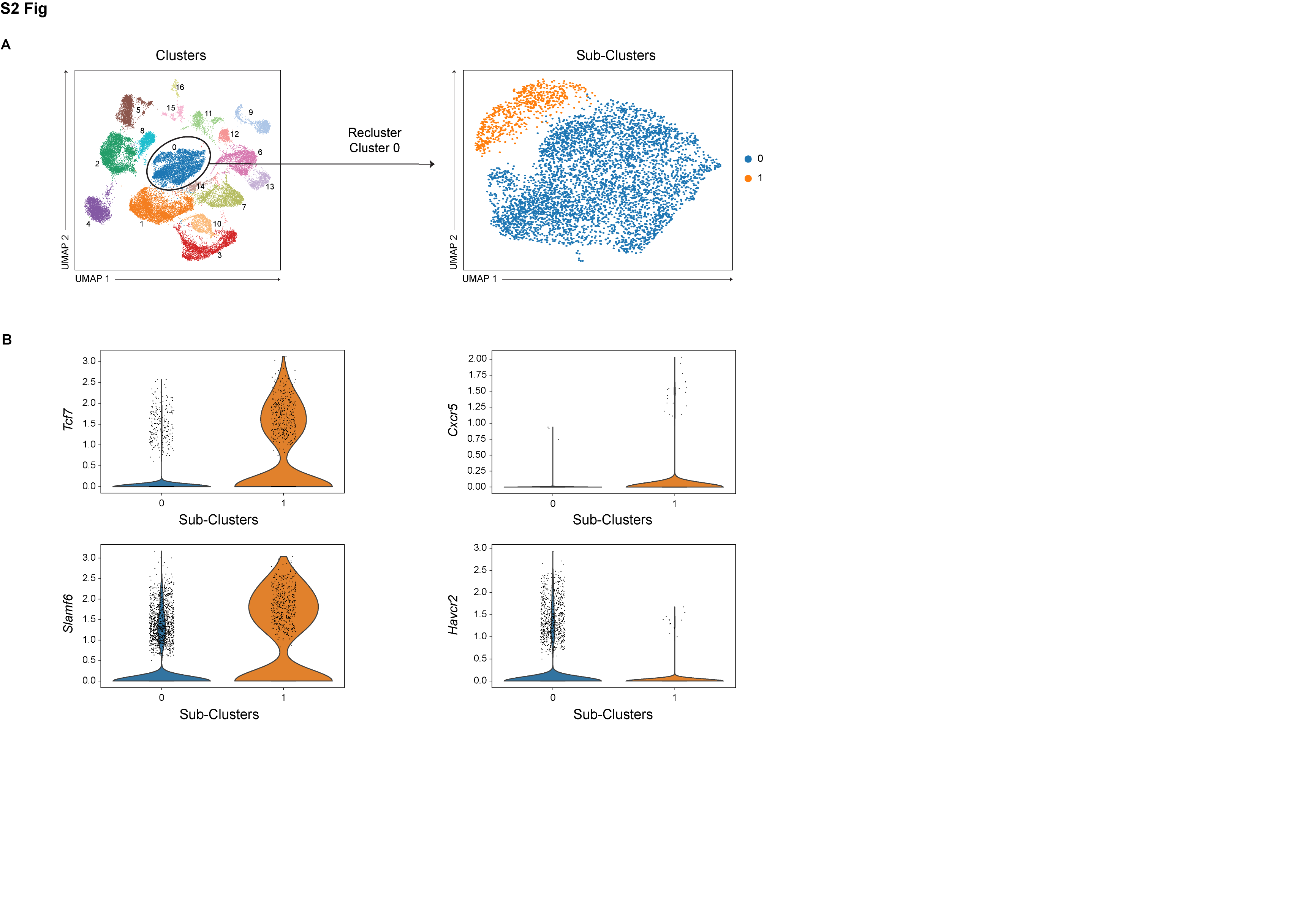

Supplement: S2 Fig — (A) Cells from Cluster 0 (see Fig 1B), which was comprised of Day 7 and Day 8 cells responding to LCMV-Cl13, were reclustered and results are presented as a new UMAP. Reclustering of Cluster 0 resulted in two subclusters (0 and 1). (B) Expression of Tcf7, Cxcr5, Slamf6, and Havcr2 by subclusters 0 and 1, represented as violin plots. Each violin represents the probability density at each value; each dot represents one cell. LCMV-Cl13, LCMV-Clone 13; UMAP, Uniform Manifold Approximation and Projection. (TIF) [file pbio.3001983.s006.tif]

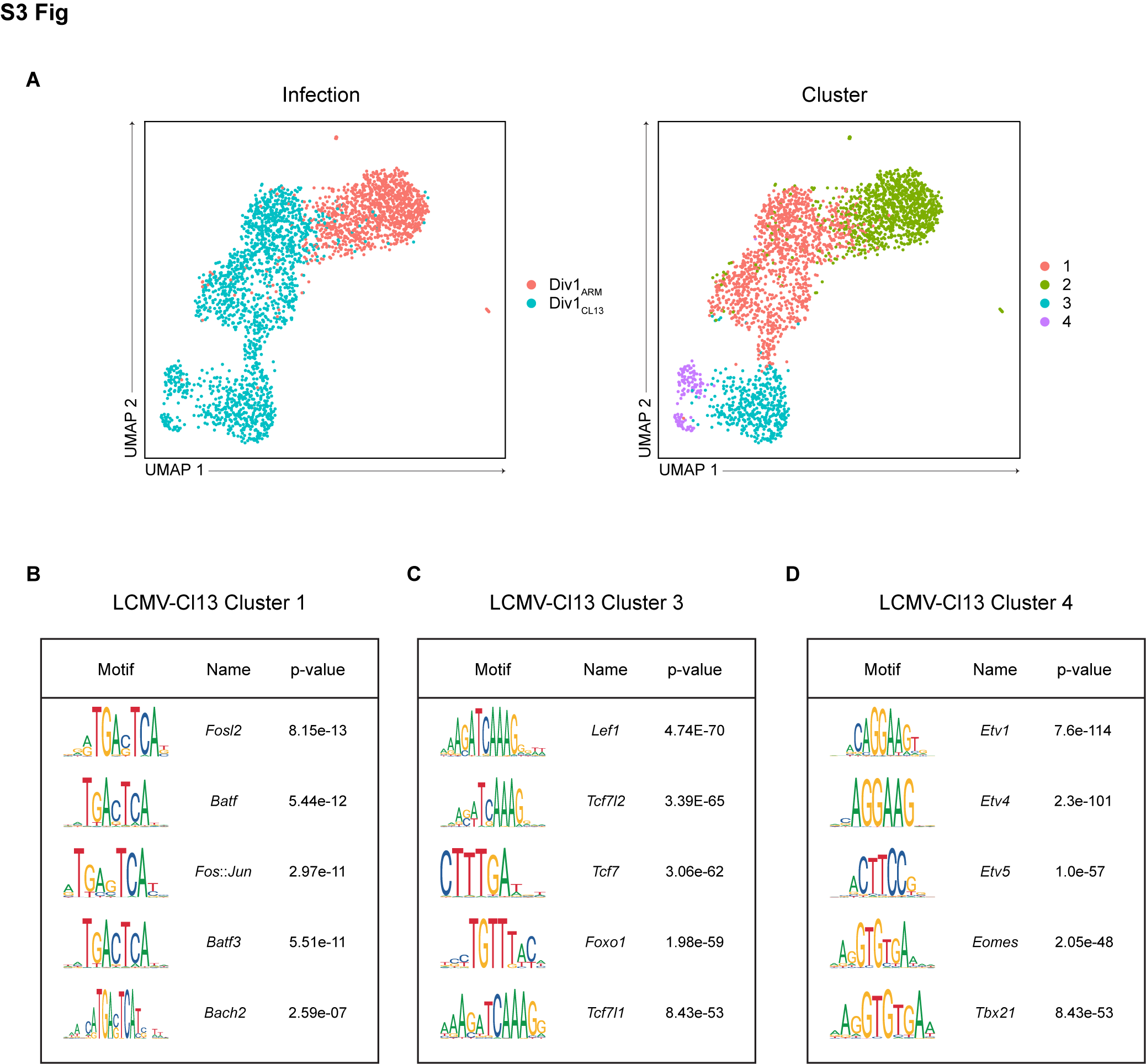

Supplement: S3 Fig — CD8+CD45.1+ P14 T cells were CSFE-labeled prior to adoptive transfer into separate CD45.2+ recipient mice that were infected with LCMV-Arm or LCMV-Cl13. Recipient mice were killed at 2 days post-infection and Division 1 (second CFSE peak) P14 T cells were FACS-isolated; nuclei were extracted and processed for scATAC-seq using the 10x Genomics pipeline. (A) UMAP clustering of all CD8+ cells on the basis of scATAC-seq data colored by infection type (left) or cluster identity (right) is shown. (B-D) Selected examples of transcription factor motifs preferentially enriched in accessible chromatin regions from each of the three Div1CL13 clusters. CFSE, carboxyfluorescein succinimidyl ester; LCMV-Arm, LCMV-Armstrong; LCMV-Cl13, LCMV-Clone 13; scATAC-seq, single-cell assay for transposase-accessible chromatin using sequencing; UMAP, Uniform Manifold Approximation and Projection. (TIF) [file pbio.3001983.s007.tif]

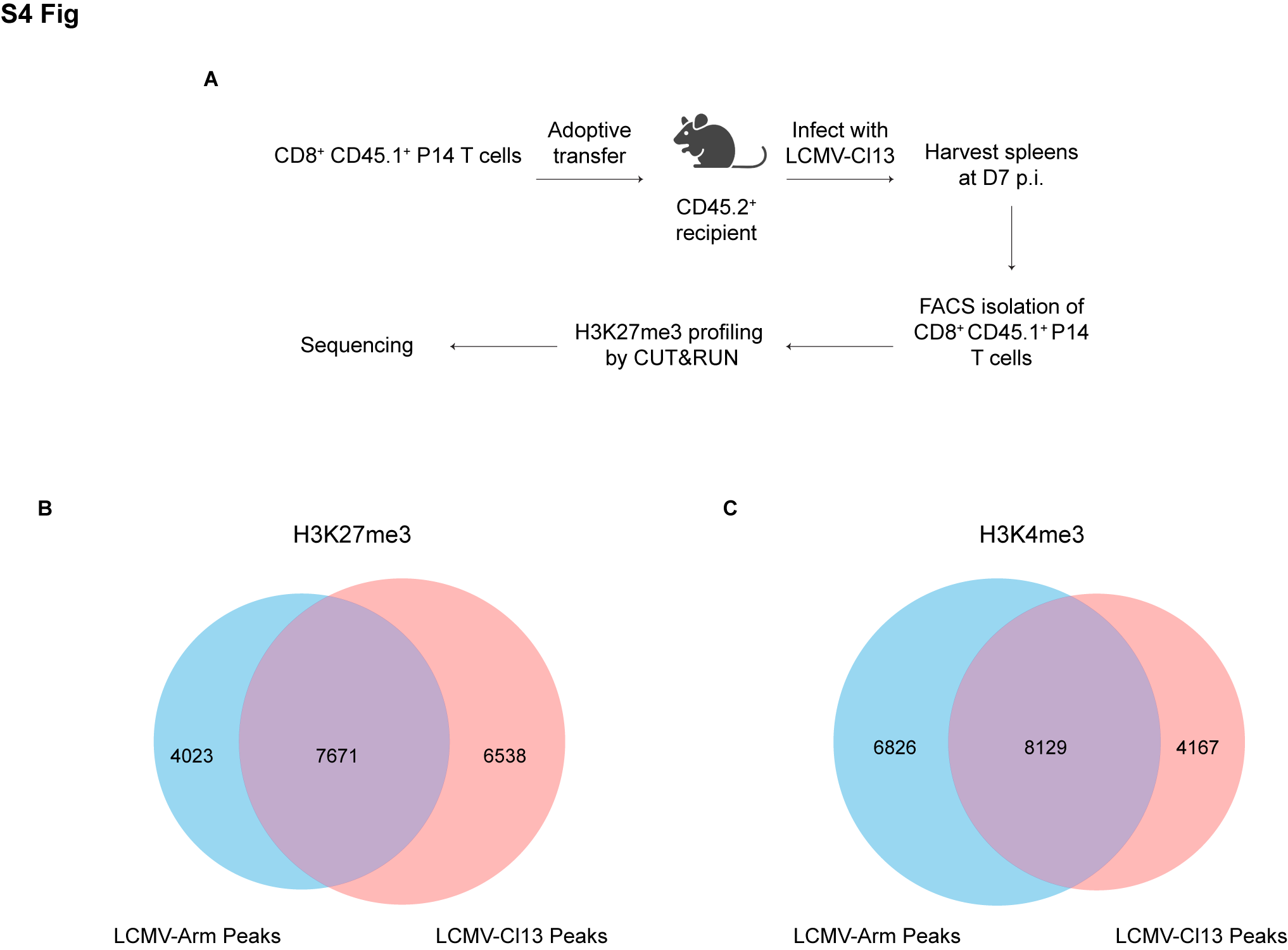

Supplement: S4 Fig — (A) Experimental setup. (B, C) Venn diagram analysis of shared and differential H3K27me3 (B) or H3K4me3 (C) peaks identified in accessible chromatin regions from CD8+ T cells responding to LCMV-Arm vs. LCMV-Cl13. The raw data for the panels in this figure are located in S1 Data file. S4A Fig created with BioRender.com. LCMV-Arm, LCMV-Armstrong; LCMV-Cl13, LCMV-Clone 13. (TIF) [file pbio.3001983.s008.tif]

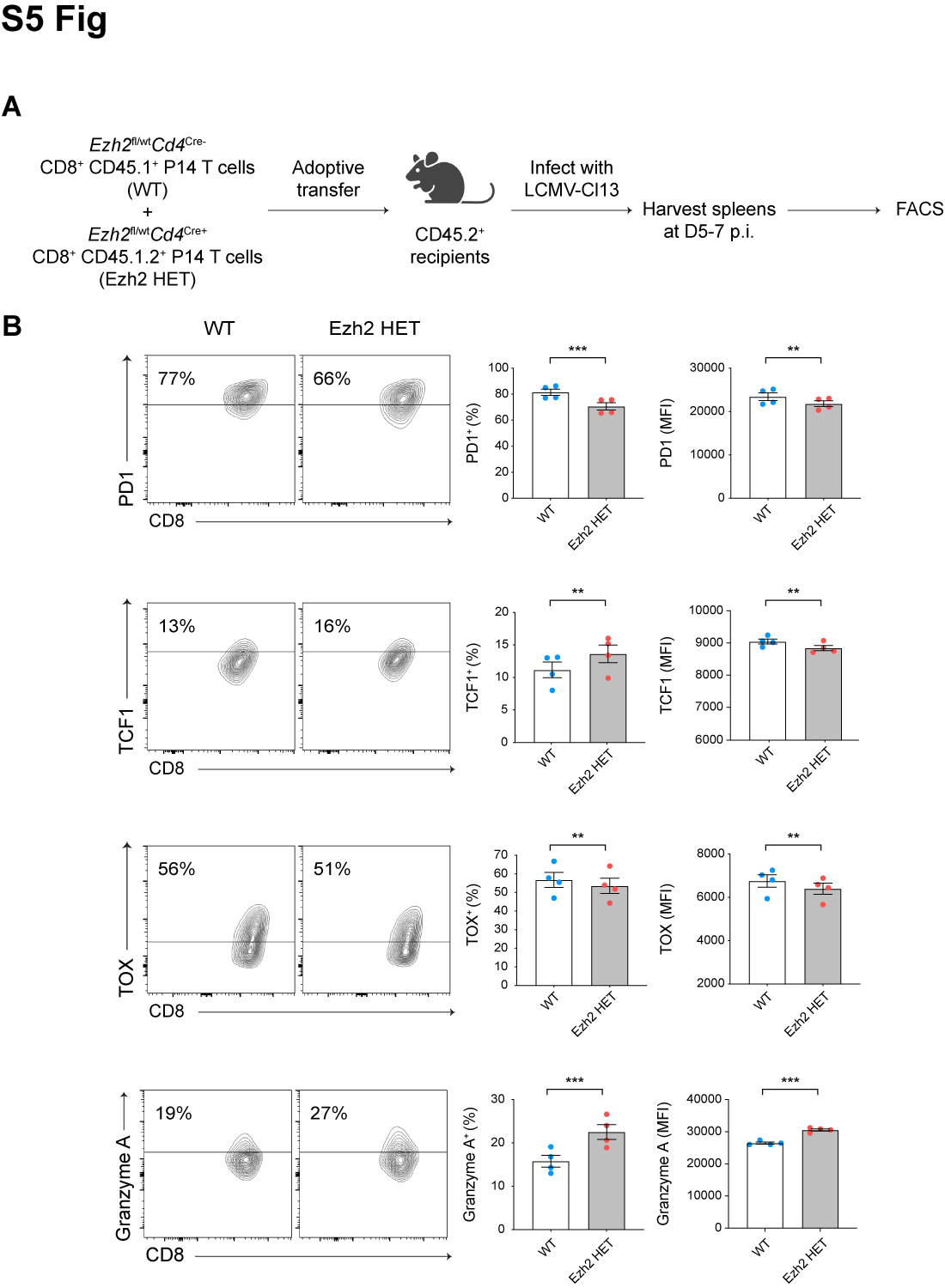

Supplement: S5 Fig — (A) Experimental setup. Control CD45.1+ (wild-type, WT) and Ezh2-heterozygous CD45.1.2+(Ezh2fl/wtCd4Cre+, Ezh2 HET) CD8+ P14 T cells were cotransferred into congenically distinct CD45.2+ recipient mice prior to infection with LCMV-Cl13; recipient mice were killed at 5–7 days post-infection and splenocytes analyzed by flow cytometry. (B) Representative flow cytometry plots (left) displaying expression of PD1, TCF1, TOX, or Granzyme A protein among gated donor WT or Ezh2 HET P14 T cells. Bar graphs indicate the frequencies (middle) or MFI (right) of WT (blue) or Ezh2 HET (red) P14 T cells responding to LCMV-Cl13. Data are shown as mean ± SEM. **p < 0.01, ***p < 0.0001 (paired t test). Data are representative of 2 to 3 independent experiments. The raw data for the panels in this figure are located in S1 Data file. S5A Fig created with BioRender.com. LCMV-Cl13, LCMV-Clone 13; MFI, mean fluorescence intensity; WT, wild-type. (TIF) [file pbio.3001983.s009.tif]

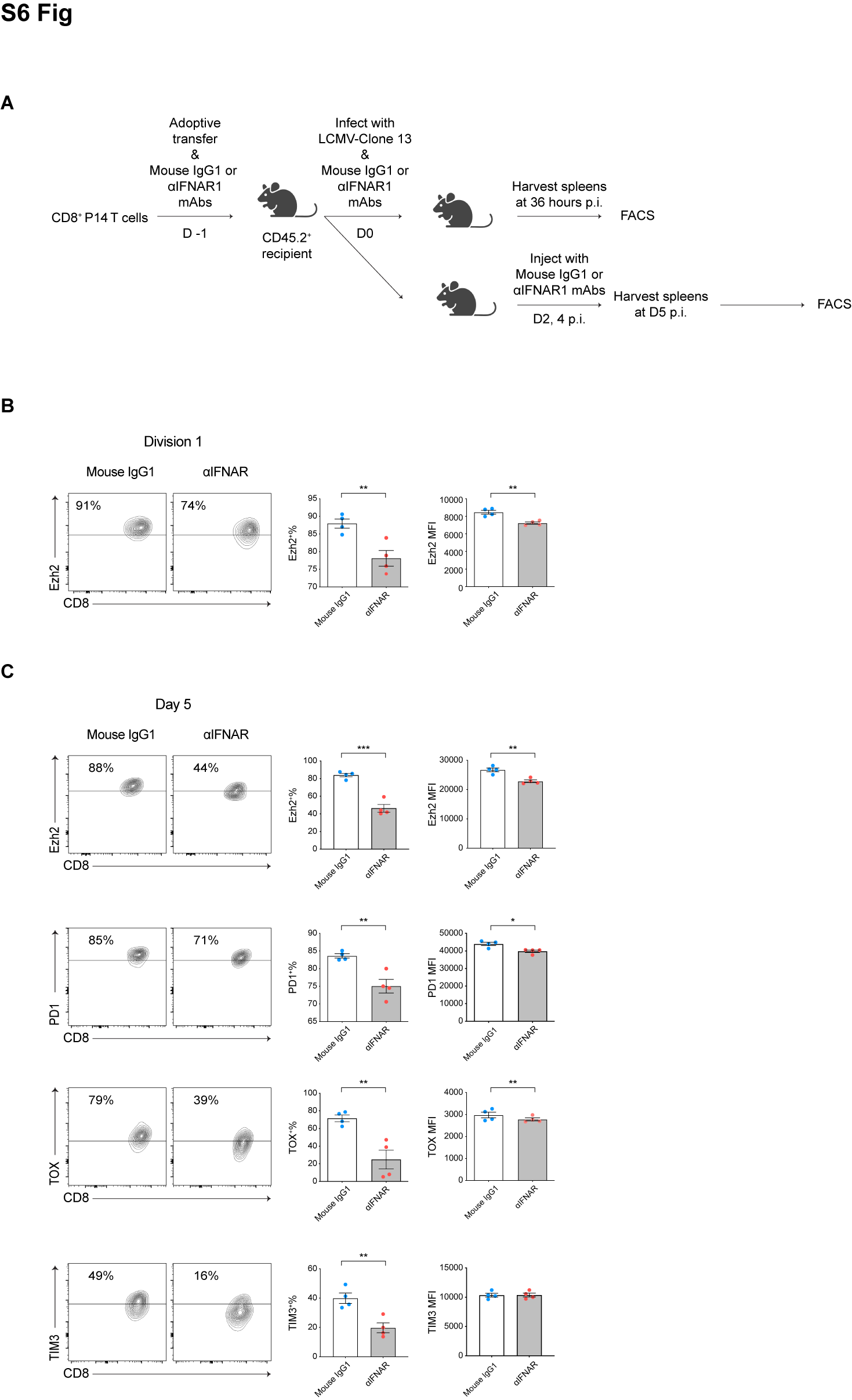

Supplement: S6 Fig — (A) Experimental setup. CD45.1+ P14 T cells were transferred into separate CD45.2+ recipient mice prior to infection with LCMV-Cl13. For analysis of Division 1 cells, P14 cells were labeled with CFSE prior to transfer. Mice were treated with control isotype mAbs or anti-IFNAR1 blocking mAbs on the day of transfer (day -1) and day of infection (day 0). For analysis performed at day 5 post-infection, antibodies were also administered on days 2 and 4 post-infection. Mice were killed on day 2 or 5 post-infection for flow cytometry analysis. (B) Representative flow cytometry plots (left) displaying expression of Ezh2 protein among gated Division 1 (second CFSE peak) isotype- vs. anti-IFNAR1-treated P14 T cells. Bar graphs indicate the frequencies (middle) or mean fluorescence intensity (MFI, right) of isotype- (blue) or anti-IFNAR1-treated (red) P14 T cells expressing Ezh2. (C) Representative flow cytometry plots (left) displaying expression of Ezh2, PD1, TOX, and TIM3 protein among isotype- vs. anti-IFNAR1-treated P14 T cells. Bar graphs indicate the frequencies (middle) or MFI (right) of isotype- (blue) or anti-IFNAR1-treated (red) P14 T cells expressing each molecule. Data are shown as mean ± SEM. *p < 0.05, **p < 0.01, ***p < 0.0001 (Student’s t test). Data are representative of 2 to 3 independent experiments. The raw data for the panels in this figure are located in S1 Data file. S6A Fig created with BioRender.com. CFSE, carboxyfluorescein succinimidyl ester; IFN-I, type I interferon; LCMV-Cl13, LCMV-Clone 13; MFI, mean fluorescence intensity. (TIF) [file pbio.3001983.s010.tif]
